# Volumetric measurement of terminal ileal Crohn’s disease by magnetic resonance enterography: a feasibility study

**DOI:** 10.1007/s00330-024-10880-8

**Published:** 2024-07-19

**Authors:** Shankar Kumar, Nikhil Rao, Anisha Bhagwanani, Thomas Parry, Maira Hameed, Safi Rahman, Heather E. Fitzke, Judith Holmes, Benjamin Barrow, Andrew Bard, Alex Menys, David Bennett, Sue Mallett, Stuart A. Taylor

**Affiliations:** 1 Centre for Medical Imaging, University College London (UCL), London, UK; 2 Department of Radiology, University Hospitals Coventry and Warwickshire NHS Trust, Coventry, United Kingdom; 3 Department of Radiology, Frimley Health NHS Foundation Trust, Frimley, United Kingdom; 4 Motilent Limited, London, United Kingdom; 5 Takeda Pharmaceuticals Limited, Cambridge, MA USA

**Keywords:** Crohn’s disease, Diagnostic imaging, Magnetic resonance enterography

## Abstract

**Objectives:**

Magnetic resonance enterography (MRE) interpretation of Crohn’s disease (CD) is subjective and uses 2D analysis. We evaluated the feasibility of volumetric measurement of terminal ileal CD on MRE compared to endoscopy and sMARIA, and the responsiveness of volumetric changes to biologics.

**Methods:**

CD patients with MRE and contemporaneous CD endoscopic index of severity-scored ileocolonoscopy were included. A centreline was placed through the terminal ileum (TI) lumen defining the diseased bowel length on the T2-weighted non-fat saturated sequence, used by two radiologists to independently segment the bowel wall to measure volume (phase 1). In phase 2, we measured disease volume in patients treated with biologics, who had undergone pre- and post-treatment MRE, with treatment response classified via global physician assessment.

**Results:**

Phase 1 comprised 30 patients (median age 29 (IQR 24, 34) years). Phase 2 included 12 patients (25 years (22, 38)). In phase 1, the mean of the radiologist-measured volumes was used for analysis. The median disease volume in those with endoscopically active CD was 20.9 cm^3^ (IQR 11.3, 44.0) compared to 5.7 cm^3^ (2.9, 9.8) with normal endoscopy. The mean difference in disease volume between the radiologists was 3.0 cm^3^ (limits of agreement −21.8, 15.9). The median disease volume of patients with active CD by sMARIA was 15.0 cm^3^ (8.7, 44.0) compared to 2.85 cm^3^ (2.6, 3.1) for those with inactive CD. Pre- and post-treatment median disease volumes were 28.5 cm^3^ (26.4, 31.2), 11 cm^3^ (4.8, 16.6), respectively in biological responders, vs 26.8 cm^3^ (12.3, 48.7), 40.1 cm^3^ (10, 56.7) in non-responders.

**Conclusion:**

Volumetric measurement of terminal ileal CD by MRE is feasible, related to endoscopy and sMARIA activity, and responsive to biologics.

**Clinical relevance statement:**

Measuring the whole volume of diseased bowel on MRE in CD is feasible, related to how biologically active the disease is when assessed by endoscopy and by existing MRE activity scores, and is sensitive to treatment response.

**Key Points:**

- *MRE reporting for CD is subjective and uses 2D images rather than assessing the full disease volume*.
- *Volumetric measurement of CD relates to endoscopic activity and shows reduced disease volumes in treatment responders*.
- *This technique is an objective biomarker that can assess disease activity and treatment response, warranting validation*.

## Introduction

Endoscopic evaluation of the gastrointestinal tract remains the gold standard for assessing Crohn’s disease (CD) activity, but it is invasive, cannot examine bowel segments proximal to the terminal ileum (TI), and may fail to detect active disease that is limited to the submucosa [1, 2]. Cross-sectional imaging, notably intestinal ultrasound (IUS) and magnetic resonance enterography (MRE) is widely implemented for diagnosing and monitoring CD, and has utility in assessing disease activity and treatment response [2–5]. The ability of multisequence MRE to act as a biomarker of disease activity has been extensively validated against endoscopic, histological and biochemical standards of reference, and various activity scores have been proposed including the simplified magnetic resonance index of activity (sMARIA) and London scores [6–13].

However, these scoring systems are rarely used in clinical practice, and MRE interpretation remains subjective with moderate interobserver agreement [14]. Bowel wall thickness is a key parameter in assessing CD activity, but measurement is undertaken using a single 2D image of the bowel, rather than assessing the full disease volume [13]. This “single slice measurement” approach forms the basis of all validated MRE activity scores. By way of comparison, in cancer imaging, it is the equivalent of measuring tumour diameter rather than tumour volume [15]. Volumetric evaluation of disease burden is common in many areas of radiology such as lung nodule assessment and whole-body MRI evaluation of multiple myeloma [16–19]. Measurement of tumour volumes also improves Response Evaluation Criteria In Solid Tumours (RECIST)-based response assessments in oncology as tumour volume may be more reproducible than linear diameter, and more sensitive for detecting tumour therapeutic response [20–22].

There is increased interest in achieving transmural healing in CD as an alternative to mucosal healing given its association with improved long-term patient outcomes [23, 24]. Cross-sectional imaging is fundamental to evaluating transmural healing and full volumetric assessment of the bowel wall may potentially be more robust than limited 2D evaluation.

Although IUS is increasingly utilised for monitoring CD and treatment response, it primarily involves the assessment of bowel wall thickness together with associated local mural parameters, and does not currently readily provide the opportunity to assess volumetric burden [25, 26].

To date, volumetric assessment of CD activity by MRE for has not been considered, although it may represent a more responsive imaging biomarker than conventional methodology. Accordingly, this study aimed to (1) evaluate the technical feasibility of volumetric measurement of TI CD on MRE, (2) explore the relationship between volume and underlying disease activity, and (3) assess whether volumetric changes in disease burden could potentially reflect response induced by biologic therapy.

## Materials and methods

Ethical permission was granted by the UCLH Research Ethics Committee (ref 10/H0720/91).

**Fig. 1:**
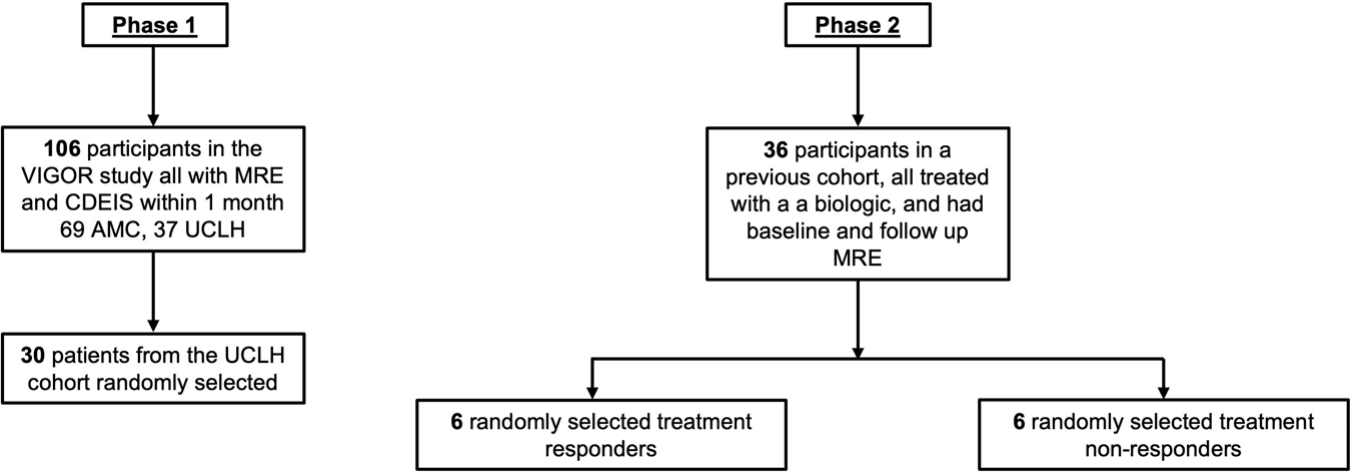
Study population. AMC, Amsterdam Medical Centre; CDEIS, Crohn’s disease endoscopic index of severity; MRE, magnetic resonance enterography; UCLH, University College Hospitals NHS Foundation Trust; VIGOR, virtual gastrointestinal tract

The study was divided into two phases (Fig. 1). Firstly, we assessed the technical feasibility and inter-observer agreement for quantifying the volumetric burden of TI CD on MRE. We also explored the relationship between the volumetric evaluation and disease activity against both an endoscopic score, and the sMARIA. In the second phase, we measured the pre- and post-treatment volumetric burden of CD in a subset of treatment responders and non-responders from a prior study validating the magnetic resonance enterography global activity score [27].

### Phase 1

We retrospectively identified the first 30 consecutive patients recruited from our hospital site to a previously reported study developing semi-automated measurements of MRI wall thickness and contrast enhancement [28]. In brief, patients aged ≥ 18 years with suspected or known CD and prospectively underwent both MRE (using a standard MRE protocol, Appendix 1) and ileocolonoscopy within 2 weeks. The Crohn’s disease endoscopic index of severity (CDEIS) was prospectively recorded in the TI as part of the study protocol [29]. Exclusion criteria included contraindication to MRE, a final diagnosis that was not CD, inability to adhere to the oral contrast protocol, a time interval of more than 2 weeks between MRE and ileocolonoscopy, incomplete MRI protocol/images of insufficient quality, and inadequate bowel cleansing that precluded accurate mucosal assessment by the endoscopist.

**Fig. 2:**
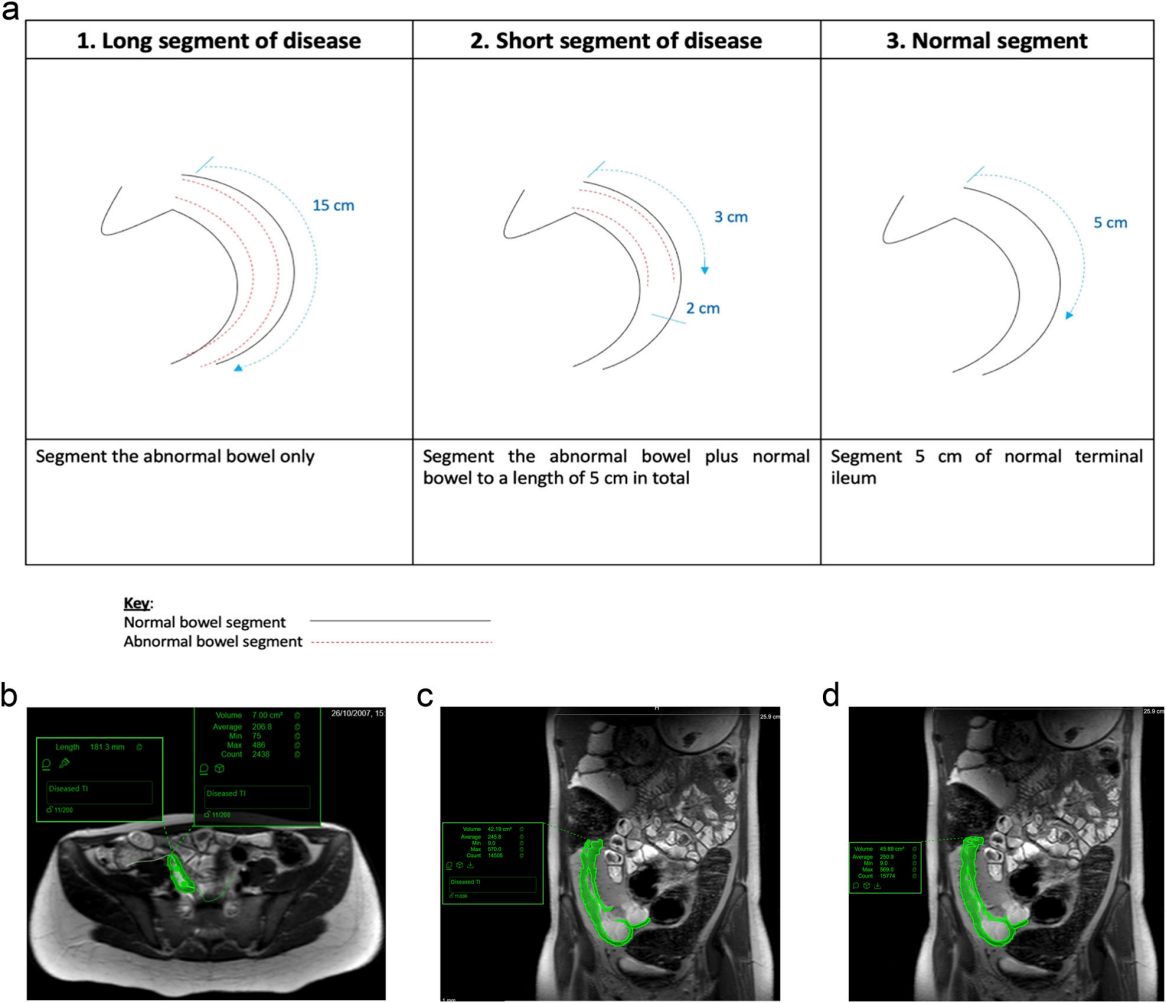
**a** Schematic and (**b**) example of how the centreline and segmentation placement were performed, and an example case with segmentation performed by (**c**) Reader 1 and (**d**) Reader 2. TI, terminal ileum

The anonymised MRE studies were uploaded to the online platform Entrolytics (Motilent, London, UK) for volumetric analysis. Using a software annotation tool, a board-certified abdominal radiologist with 7 years of experience in MRE (Reader 1) placed centrelines through the lumen of the TI that defined in their opinion the full length of diseased bowel on the T2-weighted non-fat saturated sequence. The radiologist could choose to use either the coronal or axial sequence based on which they felt it was easiest to place the centrelines to reflect the full extent of the disease. Access to all other MRE sequences was permitted to aid in the evaluation of the disease length. A single radiologist placed these centrelines to remove the subjectivity of defining the length of active disease, as the primary aim was to assess the interobserver variability of volume assessment. If there was no disease apparent on MRE, a centreline through the last 5 cm of “normal” TI was drawn. In cases where there was less than 5 cm of diseased TI, a second polyline was placed that started from the end of the diseased segment that included normal TI so that the total length was 5 cm (including diseased and normal TI) (Fig. 2a). These centrelines were used as the basis for manual segmentations of the involved bowel wall, performed independently by two board-certified abdominal radiologists (one of whom had placed the original centreline i.e. Reader 1, and another with 6 years of experience of MRE, Reader 2). They aimed to capture the whole disease volume over the length of the centrelines, manually segmenting all pixels within the bowel wall from mucosa to serosa, using a painting tool. The bowel lumen and adjacent structures such as fat and vessels were not included (Fig. 2b). Both readers performed this segmentation independently using the same centrelines (Fig. 2c, d). The first reader also derived the sMARIA for each patient [8]. Reader 1 also recorded the time taken to apply the centreline, as well as the associated segmentation.

A secondary objective was to assess the interobserver variability of volume assessment if both the centreline application and segmentation were performed independently. Using the same methodology as described, a board-certified abdominal radiologist with 11 years of experience in MRE (Reader 3) placed a centreline and performed segmentation independently. We compared the interobserver agreement between Reader 1 and Reader 3.

### Phase 2

For this component of the study, we randomly selected data from 12 patients recruited from a previous study validating an MRE activity score [27]. In summary, this group comprised patients aged ≥ 14 years with CD starting either infliximab (Remicade, Schering-Plough) or adalimumab (Humira, AbbVie) for active CD who had a baseline MRE examination within 3 months of starting anti-TNFα therapy (up to 2 months before or 1 month after), and at least one follow-up MRE examination no earlier than 3 months after baseline. A physician’s global assessment incorporating all available clinical information was used to categorise patients as treatment ‘responders’ or ‘non-responders’ as per the original publication [27]. This was undertaken by a board-certified Consultant Gastroenterologist with a subspecialty interest in IBD and 15 years of specialist clinical experience. They were blinded to the imaging results but considered all other available clinical data including all inpatient episodes, clinic letters, endoscopy, and blood and stool results such as CRP and calprotectin. Patients were classified into one of four categories: “remission” = lack of gastrointestinal symptoms and normal CRP; “mild” = ambulatory patient, eating and drinking, no significant weight loss, lack of fever, obstruction, mass or tenderness and CRP increased above the upper limit of normal; “moderate” = intermittent vomiting or weight loss, ineffective treatment for mild disease, tenderness or mass but no overt obstruction, raised CRP; “severe” = severe weight loss or obstruction or abscess, persistence of symptoms despite intensive treatment and increased CRP at time points corresponding to each MRE [30]. Patients were defined as clinical responders if their clinical status improved by at least one rank along the scale of disease activity (e.g. by moving from moderate to mild), between the pre-treatment and post-treatment MRE study. Similarly, patients were defined as clinical non-responders if their clinical status worsened by at least one rank along the scale of disease activity (e.g. by moving from moderate to severe), between the pre-treatment and post-treatment MRE study. Exclusion criteria included insufficient data to perform global physician assessment at either baseline or at follow-up, no demonstrable CD on the baseline MRE, had undergone bowel surgery in the preceding 6 months, did not receive intravenous contrast material for their MRE, or had perianal disease.

**Fig. 3:**
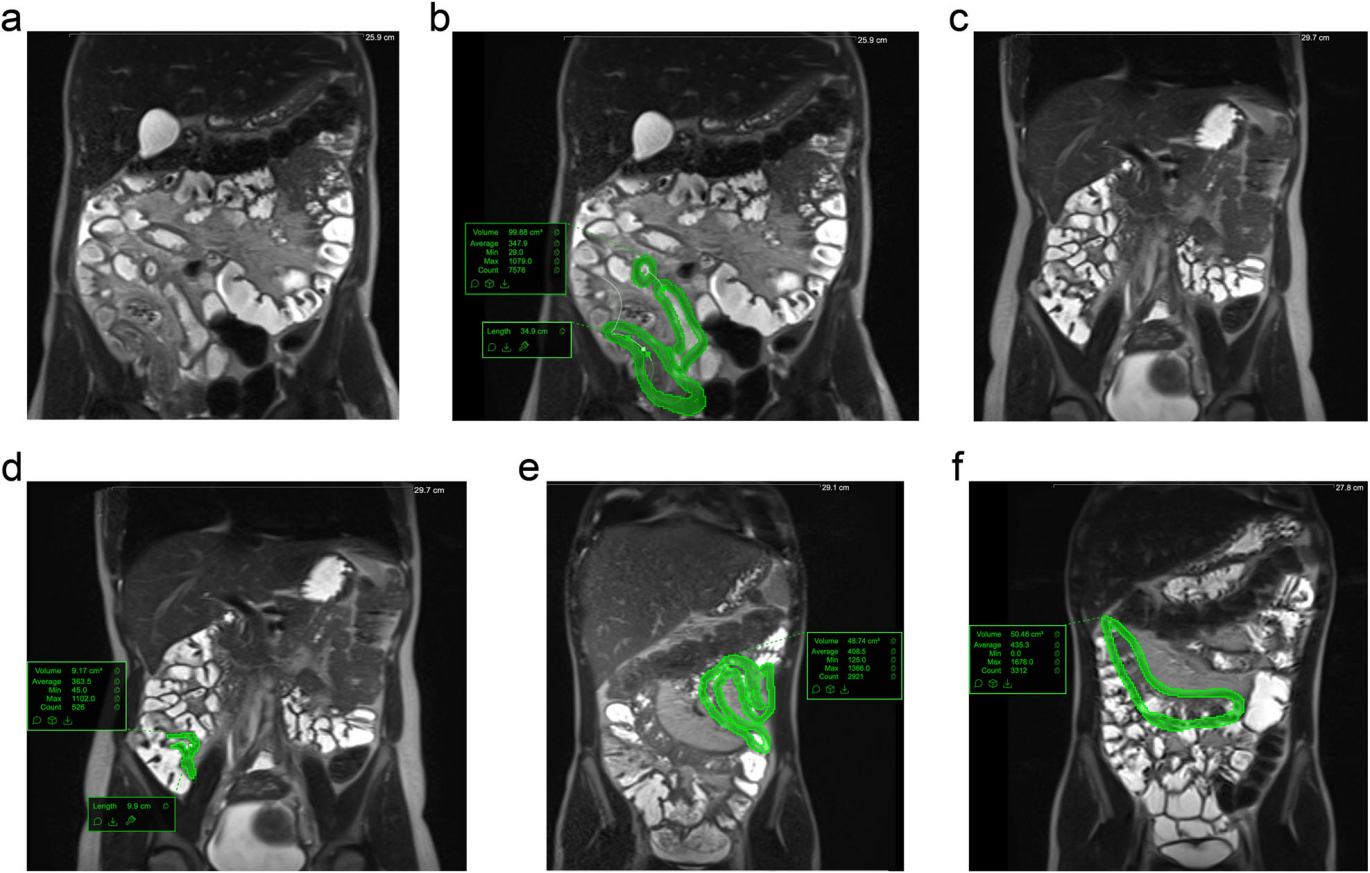
Example of a treatment responder in phase 2 showing their (**a**) pre-treatment (not annotated), (**b**) pre-treatment (annotated), (**c**) post-treatment (not annotated), and (**d**) post-treatment (annotated) MRE. An example of a treatment non-responder in phase 2 is showing their (**e**) pre-treatment and (**f**) post-treatment MRE. Some areas of disease that are apparent on these figures have been segmented and captured on alternative slices (not depicted here)

For the present study, for a proof of concept, we randomly selected six treatment responders and six non-responders who had CD. A board-certified abdominal radiologist with 11 years of experience in MRE placed a centreline that defined the length of diseased bowel on the T2-weighted non-fat saturated sequence including segments other than the TI. Again, they selected either the axial or coronal sequence dependent on which they considered to best represent the CD burden. They used this centreline as the basis for manual segmentations of the involved bowel wall using the same methodology as described above for both the pre- and post-treatment MRE studies, blinded to the treatment response classification. Examples are shown in Fig. 3. The sMARIA was also derived on the pre- and post-treatment scan from each patient, reflective of a currently utilised method that incorporates wall thickness.

### Statistical analysis

The primary outcome was the difference in pre- and post-treatment disease volumes in responders and non-responders (phase 2). Secondary outcome #1 was the difference in mean disease volume in active and inactive patients. We used the mean disease volume of the two reader measurements, and defined active disease by endoscopy as CDEIS ≥ 3, and by MRE as sMARIA ≥ 1 [8, 31]. Secondary outcome #2 (phase 1) was the agreement of disease volume measurements made if the same centreline was used for the basis of segmentation (Reader 1 vs Reader 2) and if both the centreline and segmentation were performed independently (Reader 1 vs Reader 3).

We used descriptive statistics and data visualisation to address the outcomes of our study. We used the Wilcoxon matched-pairs signed-rank test to assess the null hypothesis that the distribution of pre- and post-treatment disease volumes was different, by responder type. We did not interpret the *p*-values generated by the test because of the negligible statistical power to detect any significant differences that can be generalised to a clinical population. This is a preliminary proof of concept feasibility study to generate estimates for a future study, where a sample size calculation would be appropriate to evaluate clinical efficacy. All data analysis was conducted using Stata 18 (StataCorp, 2023).

## Results

### Phase 1

**Fig. 4:**
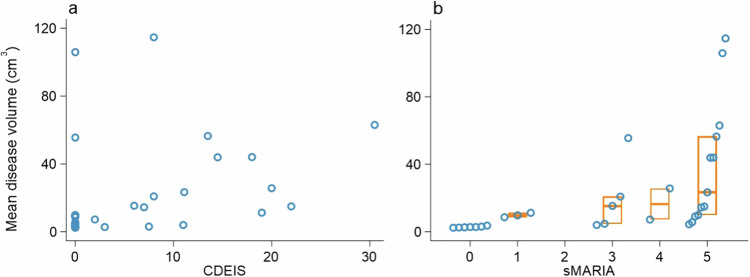
Per-patient mean disease volume against (**a**) CDEIS and (**b**) sMARIA. Orange boxes represent the median and IQR. CDEIS, Crohn’s disease endoscopic index of severity; sMARIA, simplified magnetic resonance index of activity

We studied 30 patients in the first phase of this study. Their demographic and disease characteristics are presented in Table 1. The median disease volume in those with endoscopically active CD was 20.9 cm^3^ (IQR 11.3, 44.0) compared to 5.7 cm^3^ (2.9, 9.8) with normal/inactive endoscopy (Table 2). The median disease volume of patients with active CD by sMARIA was 15.0 cm^3^ (8.7, 44.0) compared to 2.85 cm^3^ (2.6, 3.1) for those with inactive CD. The relationship of mean disease volume with CDEIS and sMARIA is presented in Fig. 4. The Spearman’s rank correlation coefficient of mean disease volume against CDEIS was 0.54 (bootstrapped 95% CI 0.24, 0.84).

**Table 1:** Demographic and disease characteristics of patients in phase 1

Characteristic	Patients
	*n* = 30
Age (years)	29 (24, 34)
Female	18 (60)
Reader 1 disease volume (cm^3^)	10.1 (3.0, 24.5)
Reader 2 disease volume (cm^3^)	10.2 (4.3, 28.1)
Mean disease volume (cm^3^)	9.9 (4.1, 25.7)
CDEIS	2.5 (0.0, 11.1)
CDEIS ≥ 3	15 (50)
sMARIA	4 (1, 5)
sMARIA ≥ 1	23 (77)
Wall thickness (mm)	6 (4, 11)
Wall thickness ≥ 3 mm	23 (77)
Oedema	20 (67)
Fat stranding	18 (60)
Ulceration	15 (50)

Data are *n* (%) or median (IQR)

**Table 2:** R1 (Reader 1), R2 (Reader 2), and mean disease volumes by CDEIS and sMARIA data are *n* or median (IQR)

		*n*	R1 disease volume, (cm^3^)	R2 disease volume, (cm^3^)	Mean disease volume, (cm^3^)^a^
All patients		30	10.1 (3.0, 24.5)	10.2 (4.3, 28.1)	9.9 (4.1, 25.7)
CDEIS	< 3	15	5.4 (2.6, 10.2)	7.3 (3.3, 10.1)	5.7 (2.9, 9.8)
	≥ 3	15	18.7 (10.1, 56.0)	22.5 (12.5, 45.9)	20.9 (11.3, 44.0)
sMARIA	< 1	7	2.4 (1.8, 2.6)	3.3 (2.8, 4.3)	2.8 (2.5, 3.1)
	≥ 1	23	13.5 (7.0, 45.3)	16.5 (9.1, 45.9)	15.0 (8.6, 44.0)

^a^ Mean volume of the two readers. The median (IQR) of the mean disease (R1 volume + R2 volume/2) of the patients is presented

**Fig. 5:**
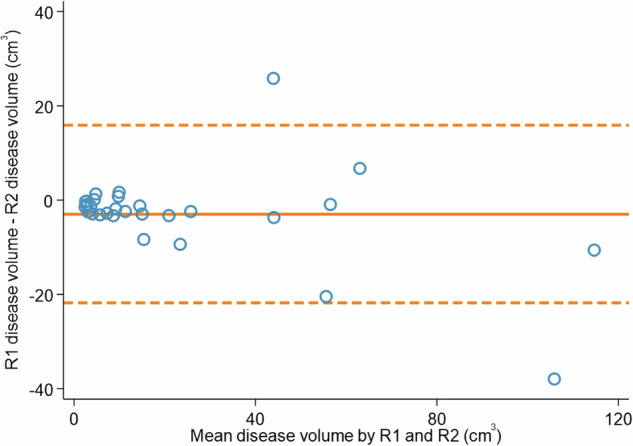
Bland–Altman analysis of R1 and R2 disease volumes. The solid line represents a mean difference of −3 cm^3^ and dashed lines represent limits of agreement from −21.8 cm^3^ to 15.9 cm^3^

**Fig. 6:**
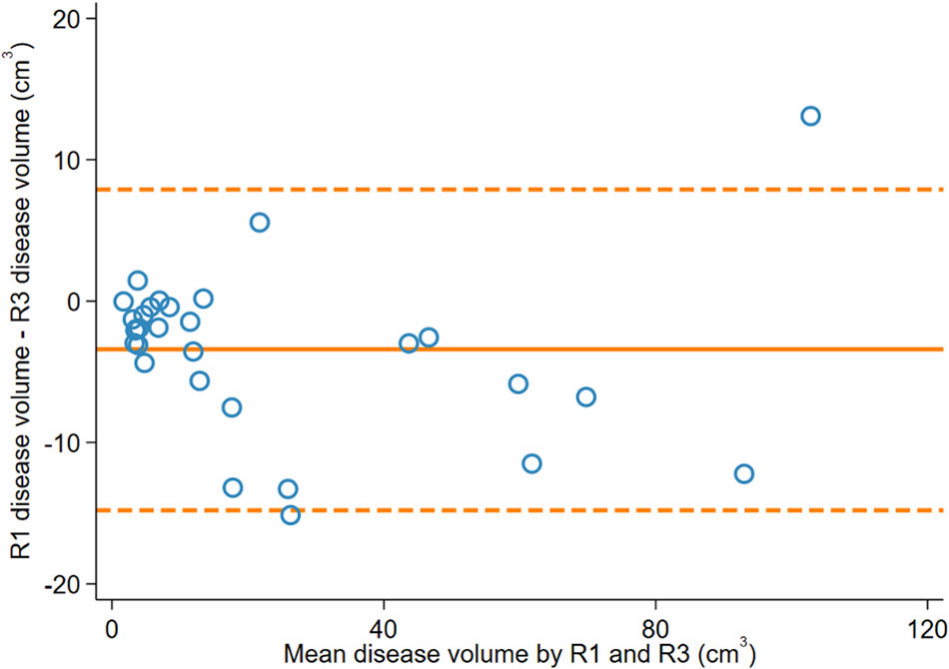
Bland–Altman analysis of R1 and R3 disease volumes. The solid line represents a mean difference of −3 cm^3^ and dashed lines represent limits of agreement from −14.8 cm^3^ to 7.9 cm^3^

The mean difference in disease volume between Reader 1 and Reader 2 was −3 cm^3^ (LoA −21.8, 15.9), and the corresponding Bland–Altman plot is presented in Fig. 5. The mean difference in disease volume between Reader 1 and Reader 3 was −3 cm^3^ (LoA −14.8, 7.9), and the corresponding Bland–Altman plot is presented in Fig. 6. The median time taken to place the polyline was 4 min and 41 s (IQR 3:11, 6:23), and a median duration of 7 min and 50 s (IQR 7:50, 35:46) to segment the bowel wall (Table 3).

**Table 3:** Time taken to draw the polyline and segment the bowel wall

Time taken (min:s)	Drawing of polyline	Segmentation of the bowel wall
	*n* = 30	*n* = 30
Median	4:41	16:12
Inter-quartile range	3:11, 6:23	7:50, 35:46
Range	1:32, 10:31	2:38, 57:29
Mean	4:50	20:18
Standard deviation	2:07	15:19

### Phase 2

**Fig. 7:**
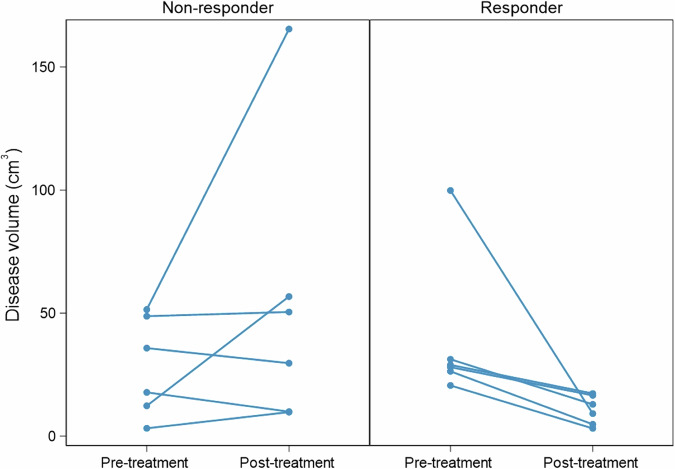
Per-patient pre-treatment and post-treatment volumes by responder type

This phase comprised 12 patients. Patient demographics and clinical details are provided in Table 4. Responders had a median disease volume of 28.5 cm^3^ (IQR 26.4, 31.2) on the pre-treatment scan and 11 cm^3^ (IQR 4.8, 16.6) on the post-treatment scan. The median of the differences between pre- and post-treatment disease volumes among responders was −17.9 cm^3^ (IQR −21.5, −11.6). The non-responders had a median disease volume of 26.8 cm^3^ (IQR 12.3, 48.7) on the pre-treatment scan and 40.1 cm^3^ (IQR 10, 56.7) on the post-treatment scan (Table 5). The median of the differences between pre- and post-treatment disease volumes among non-responders was 4.2 cm^3^ (IQR −6.1, 44.4). The disease volume changes for responders and non-responders are shown in Fig. 7.

**Table 4:** Demographic and disease characteristics of responders and non-responders

Characteristic		Non-responder	Responder	Total
		*n* = 6	*n* = 6	*n* = 12
Age		30 (23, 42)	24 (21, 29)	25 (22, 38)
Female		2 (33)	2 (33)	4 (33)
Biologic	Adalimumab	5 (83)	3 (50)	8 (67)
	Infliximab	1 (17)	3 (50)	4 (33)
Pre-existing steroids		1 (17)	3 (50)	4 (33)
Pre-existing immunosuppressant at the time of biologic	Azathioprine	3 (50)	5 (83)	8 (67)
	Methotrexate	1 (17)	0 (0)	1 (8)
	None	2 (33)	1 (17)	3 (25)
Switch from infliximab		1 (17)	0 (0)	1 (8)
Days from MRE to biologic		−38 (−51, −13)	2 (−21, 19)	−17 (−38, 4)
Surgical history	Yes	3 (50)	2 (33)	5 (42)
Montreal A	A1	1 (17)	1 (20)	2 (18)
	A2	5 (83)	4 (80)	9 (82)
Montreal L	L3	5 (83)	3 (50)	8 (67)
	L3 + L4	1 (17)	3 (50)	4 (33)
Montreal B	B1	0 (0)	3 (50)	3 (25)
	B2	4 (67)	2 (33)	6 (50)
	B2 + P	1 (17)	0 (0)	1 (8)
	B3	1 (17)	0 (0)	1 (8)
	B3 + P	0 (0)	1 (17)	1 (8)

Data are *n* (%) or median (IQR)

*A* age, *B* behaviour, *L* location, *P* perianal

**Table 5:** Difference in pre-treatment and post-treatment disease volumes by responder type

Responder type	Pre-treatment disease volume, (cm^3^)	Post-treatment disease volume, (cm^3^)	Difference in disease volume (post–pre), (cm^3^)	*p*-value
Non-responder	26.8 (12.3, 48.7)	40.1 (10.0, 56.7)	4.2 (−6.1, 44.4)	0.438
Responder	28.5 (26.4, 31.2)	11 (4.8, 16.6)	−17.9 (−21.5, −11.6)	0.031

Data are medians (IQR)

On a per-patient basis, all six responders had a reduction in disease volume post-treatment compared to two non-responders. The change in sMARIA for responders and non-responders is presented in Supplementary Fig. 1. Five treatment responders had a reduction in the sMARIA on the post-treatment MRE, as did four treatment non-responders i.e. four false positives compared to the physician’s global assessment (Supplementary Table 1).

## Discussion

The present study has demonstrated that volumetric measurement of CD activity can be reliably measured by MRE, with acceptable reproducibility between radiologists, and there is a relationship to CDEIS and sMARIA, suggesting that it may be a clinically useful parameter in the assessment of disease activity. In a proof-of-concept study, we also examined a group of patients with CD who had been treated with biologics, undergone paired pre- and post-treatment MRE, and were classified as either treatment responders or non-responders by their treating gastroenterologist, based on all available clinical data (global physician assessment). We found that the volumetric burden of CD was reduced in treatment responders between their pre- and post-treatment MRE, and did not in patients deemed to be clinical non-responders.

MRE is a key investigation for assessing CD activity and influences clinical decision-making [2, 9, 11, 32]. Nevertheless, current reporting is primarily based upon the assessment of a subjectively chosen isolated 2D segment of the bowel wall based on the opinion of the radiologist as to where the most active disease is within the abnormal segment [33]. This methodology also forms the basis of validated MRE activity scores [6, 8, 10]. Objective measurement of the total volumetric burden of active CD is an attractive proposition as it is inherently more comprehensive and robust than subjective selection and measurement of what appears to be the worst affected area of the bowel. Indeed, this principle has been applied to many areas of imaging, especially so in oncology where volumetric measurement of tumour burden is routinely undertaken. As interest in achieving transmural healing grows, true volumetric assessment of the bowel wall is an attractive parameter to capture.

To the best of our knowledge, this is the first study to evaluate the potential to assess the volumetric burden of active CD by MRE, and whether it is sensitive to changes induced by biologic therapy. Another study quantified volume on MRE in the setting of CD, but it focused exclusively on perianal fistulae [34]. Others have considered the feasibility of automatic detailed quantitative assessment of small intestinal motility in abdominal 3D cine-MRI but without comparison to clinical markers [35]. We feel our work represents an important first step in exploring more objective and robust means of exploiting MRE to accurately quantify disease activity. We confirmed that interobserver variability is acceptable even if the application of the centreline and segmentation are performed independently. Having determined that measuring the volume of CD by MRE is feasible and relates to the current best reference standards of CDEIS and sMARIA, the next step is to further examine the clinical utility of volumetric assessment by MRE in larger cohorts. Specifically, we will investigate whether multi-sequence volumetric quantification adds value over current assessment for defining CD activity, and response to biological therapy, including transmural healing. We recognise that manual segmentation of diseased bowel is impractical for clinical use as it is labour-intensive and time-consuming. In our study, we found that the median time to apply the polyline was 281 s (4 min, 41 s), with a further 470 s (6 min, 50 s) needed to segment the bowel wall, clearly precluding this as a clinically viable tool. For our proposed methodology to ultimately be used in clinical trials and clinical practice, automated or at least assisted segmentation would be required. The present work may inform and prioritise the development of such technology [36]. Such automated software could also be developed to quantify disease volume using cine loops from IUS.

Our work has limitations, including a small sample size, especially for the proof-of-concept component. However, it confirms the feasibility of measuring the volumetric burden of CD. We acknowledge that much larger studies are needed to confirm the clinical utility of volumetric assessment for CD over existing tools, although our data should help to prioritise this work and facilitate estimates to ensure future studies are sufficiently powered. Finally, the scans were performed at a single institution and our analysis was restricted to the TI. Further studies from other centres considering alternative bowel segments are needed to confirm the generalisability of this methodology, notwithstanding that we considered active CD with the TI in phase 2.

## Conclusions

Volumetric measurement of CD activity on MRE is feasible, relates to the endoscopic burden of disease and sMARIA, and is sensitive to changes induced by biologics. This work represents the first step towards developing an objective and responsive MRE-based volumetric score for CD.

## Supplementary information

ELECTRONIC SUPPLEMENTARY MATERIAL

## Abbreviations

CD: Crohn’s disease
CDEIS: Crohn’s disease endoscopic index of severity
IUS: Intestinal ultrasound
MRE: Magnetic resonance enterography
sMARIA: Simplified magnetic resonance index of activity
TI: Terminal ileum
