# Supplementary material for: Volumetric measurement of terminal ileal Crohn’s disease by magnetic resonance enterography: a feasibility study
Source: Eur Radiol. 2024 Jul 19;35(1):117–26. doi: 10.1007/s00330-024-10880-8 (PMC11632055; doi:10.1007/s00330-024-10880-8)
Supplement: Supplementary file 1 — ELECTRONIC SUPPLEMENTARY MATERIAL [file 330_2024_10880_MOESM1_ESM.pdf]

Volumetric measurement of terminal ileal Crohn’s disease by magnetic resonance enterography: a feasibility study
ELECTRONIC SUPPLEMENTARY MATERIAL

Eur Radiol (2024) Kumar S, Rao N, Bhagwanani A, et al.

Appendix 1

Magnetic resonance enterography imaging protocol

MRE was performed using a standardised clinical protocol on one of two static magnets: 1.5 Tesla (Avanto; Siemens Medical Systems, Erlangen, Germany) or 3 Tesla (Achieva; Philips, Best, The Netherlands). Patients fasted for 4 hours before consuming 1 to 1.5 L of 2.5-% mannitol solution over 45 min immediately before imaging. Twenty milligrams of intravenous hyoscine butylbromide (Buscopan; Boehringer Ingelheim, Ingelheim, Germany) were administered together with 0.1 mmol/kg of gadolinium (3 mL/sec injection using a power injector).

The following data sequences were acquired: coronal steady-state free precession gradient echo [SSFP GE] sequences without fat saturation, hyoscine butylbromide 20 mg intravenously [IV], axial and coronal fast spin echo [FSE] T2-weighted [T2W] sequences without fat saturation, coronal FSE T2W sequences with fat saturation, axial diffusion-weighted images [b values 50 and 600], and unenhanced coronal T1W sequences with fat saturation followed by contrast-enhanced T1W sequences with fat saturation [60-70 s post-injection].

Supplementary Figure 1: Per-patient R1 disease volume against R2 disease volume by CDEIS and sMARIA

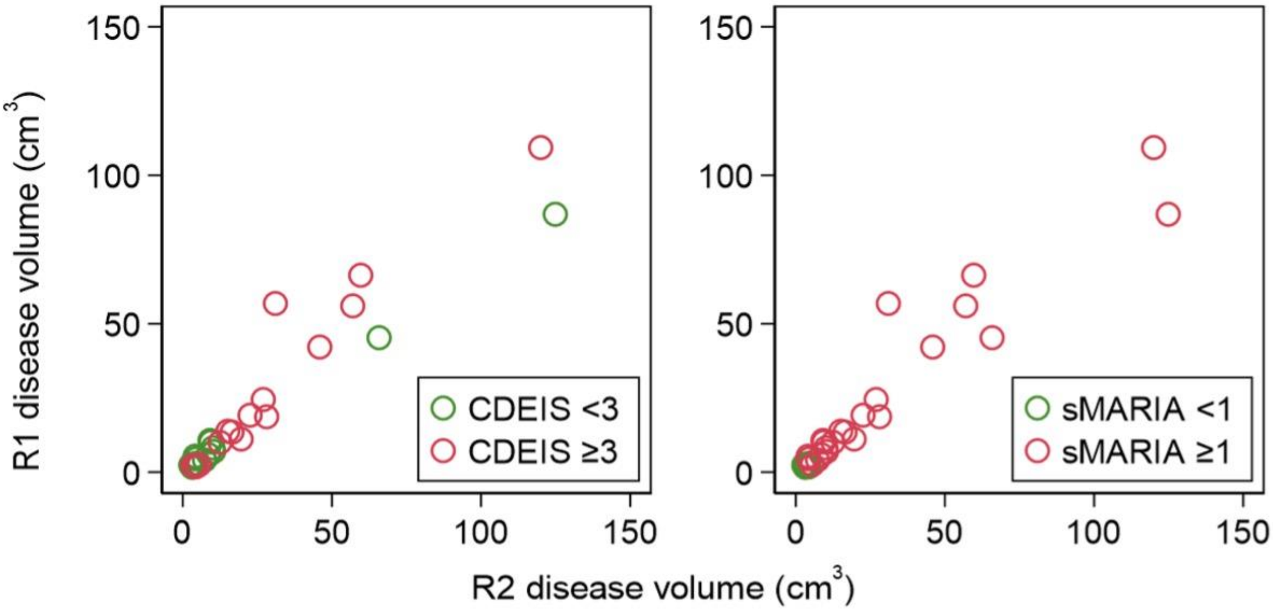

Supplementary Table 1. Pre-treatment and post-treatment sMARIA by responder type.

sMARIA		Responder type	
**Pre-treatment**	**Post-treatment**	**Non-responder n=6**	**Responder n=6**
0 (inactive)	2 (active)	1	0
1 (active)	1 (active)	0	1
2 (active)	1 (active)	1	2
2 (active)	3 (active)	1	0
3 (active)	1 (active)	1	2
3 (active)	2 (active)	2	1

Data are n
